# Training on Emotional Intelligence for Caregivers of Patients with Acquired Brain Injury and Cognitive Impairment: A Quasi-Experimental Study

**DOI:** 10.3390/ijerph192114050

**Published:** 2022-10-28

**Authors:** Irene De-Torres, Fernando Bustos, Juan Carlos Arango-Lasprilla, Pablo Fernández-Berrocal

**Affiliations:** 1 Málaga Regional University Hospital, Hospital Civil, 29010 Málaga, Spain; 2 Department of Language and Linguistics, University of Essex, Colchester CO4 3SQ, UK; 3 Department of Psychology, College of Humanities and Sciences, Virginia Commonwealth University, Richmond, VA 23284, USA; 4 Department of Basic Psychology, Faculty of Psychology, University of Málaga, 29071 Málaga, Spain

**Keywords:** emotional intelligence, brain injury, cognitive impairment

## Abstract

Background: Cognitive-behavioral alterations can occur after an acquired brain injury (ABI). Objectives: To develop and evaluate a synchronous online training program on emotional intelligence (EI) for the caregivers of adult patients with cognitive-behavioral impairment due to ABI. Methods: Quasi-experimental study. Ten caregivers attended a one-month virtual synchronous course about EI. The emotional status of the caregivers was registered one-month-previous and one-month-post program using comparative measures: The Trait Meta-Mood Scale (TMMS-24), the Positive and Negative Affect Schedule (PANAS), Caregiver Burden Interview, the 10-item Connor-Davidson Resilience Scale, and the Emotional Health Survey. Results: After the training course, the favorable changes related to emotional affect measured with the PANAS questionnaire were found; both positive (increase; Mdn = 39.5; effect size −12.79; adjusted variance 95.75) and negative (decrease; Mdn = 14.5; effect size 0.73; adjusted variance 95.50) presented a statistical significance of *p* < 0.05. The TMMS-24 post-test showed that 90% of the caregivers reported an adequate or excellent emotional repair (*p* < 0.05; effect size −0.68; adjusted variance 94.75). No other significant differences were found. Conclusions: After this training in EI, the caregivers had a more positive mood and improved aspects of their emotional intelligence, such as emotional regulation. More studies need to be conducted.

## 1. Introduction

Cognitive-behavioral alterations that occur suddenly in the life of an adult who has suffered an acquired brain injury (ABI) radically alter their quality of life and their relatives. ABI may be caused for different reasons: traumatic brain injury (TBI) or stroke. ABI can immediately reduce patients’ autonomy and their work capacity, and, therefore, it affects their economic situation. These kinds of injuries can alter their ability to communicate and relate, causing the whole family to struggle. It is a very stressful situation of great emotional complexity for the patient and their relatives.

Cognitive deficits and behavioral changes related to ABI can include slowed information processing and impaired long-term memory, working memory, attention, executive function, social cognition, and self-awareness. Mental fatigue is also frequently associated with and can exacerbate the consequences of neuropsychological deficits. Personality and behavioral changes may include combinations of impulsivity and apathy [1]. Some caregivers highlight that patients struggle with accepting criticism and recognizing when they feel offended by others [2].

Families are most affected by these behavioral changes, often in the absence of appropriate information or advice. Many caregivers report high levels of distress, burden, and depression [3]. The well-being of close relatives can be decisive for the well-being of the patient [4].

Decision-making in a home with a patient with cognitive impairment due to ABI becomes collaborative work along with the caregiver. The caregiver needs to see the patient in a positive light and learn from the experience to find a way to communicate while being committed to the relationship. Understanding the functional implications of the ABI on the patient is vital in this process [5].

Riley (2016) claimed that “some partners report that they feel abandoned and unsupported by services in the immediate aftermath of discharge, left to cope by themselves with little support or guidance at a time when their responsibilities and need for assistance have increased” [6]. For many relatives, dealing with these extra demands means that they have little time left to address their own needs [6]. Long-lasting caring for a partner with ABI presents considerable challenges that can affect the caregiver’s health and well-being. Equipping caregivers with emotional regulation strategies to provide sustainable care could benefit both the caregiver and the rehabilitation of the spouse. Caregivers’ anxiety episodes are often managed through hyper-vigilant and overprotective practices that put them at risk of burnout [7].

Emotional Intelligence (EI) refers, in general, to the ability to identify, express, and understand emotions, assimilate them into thought, and reflexively regulate one’s own and others’ pleasant and unpleasant emotions [8].

The most emotionally intelligent individuals, that is, those who possess an adequate capacity to attend to, understand, and regulate their emotions, will have better mental health, which will ultimately benefit the mental health of their partner and family [9]. A training course on EI could help caregivers improve their abilities to recognize the emotions that influence their relationship with patients, as well as better management of both verbal and non-verbal communication. Emotional regulation is the ability to influence our own emotions in relation to when we have them and how we experience and express them. Co-regulation occurs when caregivers use strategies to help their relatives regulate their emotions. Due to the sudden appearance of this difficulty, caregivers highly value training to learn how to cope with this situation in a resilient manner.

EI tools help caregivers cope with the behavioral disturbances of patients and their own unpleasant emotions in reaction to those disruptive behaviors and lack of success in managing their relatives’ behaviors that can lead to a sense of hopelessness. Some caregivers see this process as an opportunity for personal growth and development as a result of meeting the challenges posed by the injury, including a new appreciation of what life offers and the development of inner strength and resilience [6].

Some studies suggest that EI can be trained [10,11]. Other authors have designed a counseling program for caregivers of patients with TBI with objectives such as improving mutual understanding and building family consensus or shifting the perspective from negative to positive aspects of the situation and recognizing the impact of thoughts on emotions [12]. Nevertheless, there are no results about the effectiveness of these types of interventions in caregivers published in the scientific literature, and our main objective aims to answer this need.

Caregiver overload can be aggravated under challenging times, such as the situation of social distancing due to the COVID-19 pandemic. Some consequences of this health crisis are reduced social and sanitary support for patients with chronic diseases and the reduction or cessation of ambulatory therapies or day-center activities. Since the beginning of the pandemic, even attending a training course can be a challenge, so this program had to be redesigned into a virtual course.

### Objectives

To develop and evaluate a synchronous online training program on EI for caregivers of adult patients with cognitive-behavioral impairment due to ABI.

## 2. Materials and Methods

A quasi-experimental study was designed, and a target sample of ten caregivers of patients with cognitive impairment after ABI attended a one-month virtual synchronous course about EI. Inclusion criteria: adult caregivers of an adult patient with cognitive impairment after ABI. Exclusion criteria: non-cognitive impairment of the patient; aged under 18 years old; major depression or other psychiatric issues; and insufficient level of computer literacy and access to the Internet.

**Figure 1:**
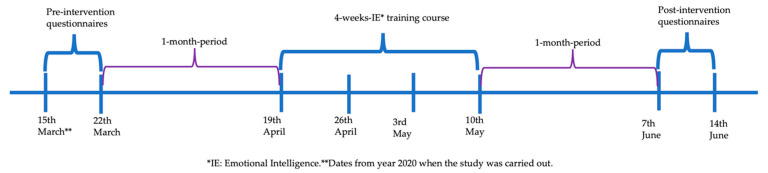
Timeline.

**Figure 2:**
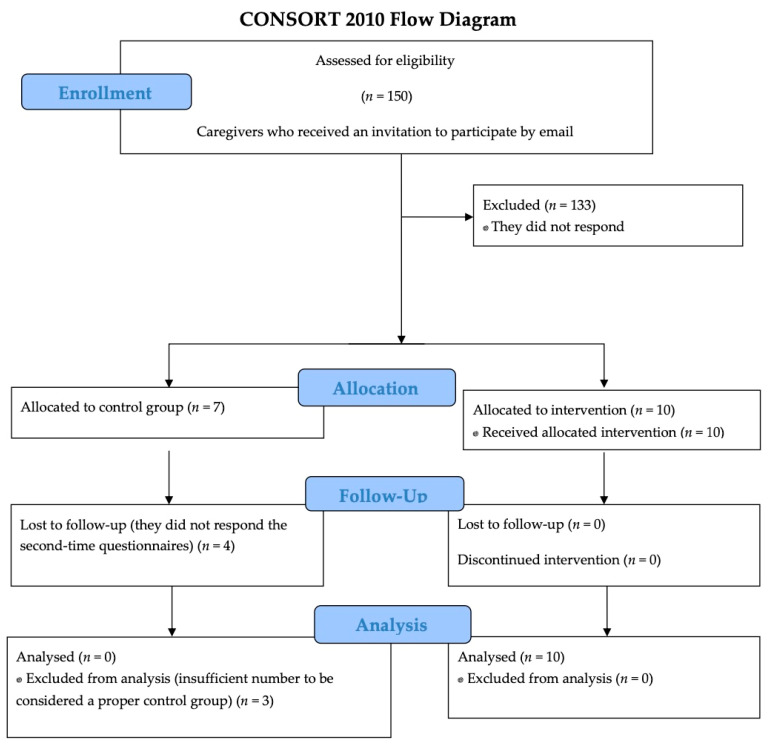
Consort Flow Diagram.

An email was sent to 150 relatives of patients with ABI and cognitive impairment, inviting them to participate in the study in March 2020 (see timeline in Figure 1). They voluntarily provided their email when accompanying the patient to the rehabilitation consultation at Hospital Regional Universitario de Málaga, Spain, for cognitive assessment to be informed of informative talks and courses on brain damage. Of the 150, 17 answered the initial questionnaires, and 10 completed the course (Figure 2). Seven of the family members who answered the initial questionnaires and did not take the course were intended to be used as a control group. However, only three of them answered the questionnaires after a period of time, so the control group was incomplete and insufficient for statistical comparisons.

The intervention program consisted of four 3 h sessions from April to May 2020. The following topics were addressed: (a) emotions’ perception and expression; (b) emotion assimilation into thought; (c) understanding of emotions; and (d) emotions’ reflective regulation. In addition to participation through a group conversation in a video call, software was used to encourage online participation, available at https://www.menti.com/ (accessed on 10 May 2020). Each week collaborative discussion about previous concepts was facilitated to relate them to emotional tools and coping strategies. Examples of daily life were used to understand key messages.

The course was designed and taught by a medical specialist in Rehabilitation with a master’s degree in Neuropsychology and Emotional Intelligence. The concepts worked on weekly are listed below:

First week: the presentation of each participant and a moment to share the case of the patient of whom they are relatives; a reflection on the usefulness of EI; a talk about the difficulties present after the hospital discharge of the patient with ABI and his family; a meditation on EI strategies they already have; situations in which we are reactive or know how to provide a more calm response; an explanation about the concept of emotion; neuroscience-based data; a description of the concept of EI and its four branches; verbal and non-verbal language; an analysis of facial expressiveness associated with emotions; emotional perception activities; basic and secondary emotions; non-verbal communication of emotions interpretation exercises; self-control activity and use of non-verbal communication; messages for home and tasks for the week of reflection through practice.

Second week: reflection on concepts learned, tasks for the week and implementation in real life; activity of perception of emotional facial expressiveness isolating parts of the face; dynamics of emotional expression and analysis; the concept of emotional facilitation; the influence of emotions on thought and behavior; emotions and attention; emotions and decision making; activity on emotion and creativity; dynamics on emotion and memory; emotion and judgment; the influence of emotions on points of view; emotion and problem solving; reflection on the perception of the emotional state of the other; examples of situations; competitive team contest on the usefulness of different emotions in various situations; use of the emotional meter by colored quadrants according to tone and degree of the emotion felt; influences posture and emotion; people strengths.

Third week: activity of the use of the emotional meter; reflection on tasks of the week; time to share changes in emotional management at home; consolidation of previous concepts; review of past classes; body language activity; understanding of emotions; simultaneous emotions; conflicting emotions; emotional escalation and transitions; meaning and labeling of emotions; examples of patient caregivers; a reflective story about the usefulness of unpleasant emotions; intra- and interpersonal functions of emotions; mixed emotions game; affective and cognitive empathy; mirror neurons; exercise “the glasses of empathy”; verbal and non-verbal aspects of empathy; balance between pleasant and unpleasant emotions; activity called “the deaf frog”; emotional agreement and negotiation; messages for home.

Fourth week: a review of previous concepts through participatory activities; time to share progress at home; reflection on the concept of emotional regulation; emotional mismanagement; cognitive and behavioral coping and distraction strategies; analysis of everyday situations; reflection through movies; consequences of emotional repression; selection or modification of situations; attentional or cognitive change; response modulation; reevaluation versus repression; proactive versus reactionary; emotional hygiene; resilience; conflict resolution techniques; metacognitive strategies; constructive feedback; analysis of strengths and weaknesses; review of achieved objectives; closing dynamics.

Through a web link, the caregivers were asked to answer several questions to achieve a complete description of the sample (i.e., age, gender, number of years as a caregiver, relationship with the patient, presence of more dependent people in their charge, cause of brain injury of the patient, primary cognitive deficit of the patient from the caregiver’s point of view), and the questions of the scales that are described below.

Comparative measures were administered to record the emotional status of caregivers one month before and after the training program (pre- and post-tests):

1. The Trait Meta-Mood Scale (TMMS-24) [13] was designed to assess relatively stable individual differences in people’s tendency to attend to their moods and emotions, discriminate clearly and regulate them. The results are divided into three dimensions: attention (I am able to feel and express feelings appropriately), clarity (I understand my emotional states well), and repair (I am able to regulate emotional states correctly). The scores are adapted to male and female populations. Each one of the three key dimensions (attention to feelings, emotional clarity, and repair and regulation of the emotions) was evaluated on a five-point Likert-type scale (1 = strongly disagree, 5 = strongly agree). Fernández-Berrocal et al. (2004) [13] obtained alphas of 0.90, 0.90, and 0.86, respectively.
2. The Positive and Negative Affect Schedule (PANAS) [14], which is one of the most used affect measures, is a 20-item self-report questionnaire. The range of punctuation is 10–50 for positive affect as well as for negative affect, 10 being the least presence of positive/negative affect in the life of the user in the previous month, and 50, the maximum. Díaz-García et al. (2020) [14] showed statistically significant pretest-posttest differences for both PANAS-Positive and PANAS-Negative, with moderate to large effect sizes, suggesting that the scale is able to detect changes in affectivity and, therefore, can be used to examine the impact of an intervention.
3. Zarit’s Questionnaire evaluates factors contributing to feelings of the burden of caregivers of patients. The score range is 0–88; fewer than 46 points correspond to an absence of overload, 46–56 show overload, and more than 56 show intense overload [15]. Yu et al. (2019) [16] showed an optimal cut-off score of 48 to distinguish the lower and higher burdens for the risk of psychological distress, with a sensitivity of 73% and a specificity of 80% for depression. Zarit’s Questionnaire is the most widely used tool for measuring the level of subjective burden among caregivers, and it has been validated across many populations of caregivers [16].
4. The 10-item CD-RISC [17] is an instrument for measuring resilience that has shown significant psychometric properties. Resilience has been defined as a dynamic process of adaptation to changes in life circumstances. The user has to answer the level of agreement in ten sentences about the previous month. The range of punctuation of this test is 0–40. Equal or fewer than 27 points is considered low resilience, 28–35 is an adequate level, and 36 or more is a high resilience capacity [18]. The 10-item CD-RISC measures are highly correlated with scores on the original 25-item CD-RISC (r = 0.92) [18].
5. MH5 is part of the Short Form-36 Health Survey [19]. It is one of the most widely used and evaluated generic health-related quality of life questionnaires. MH5 is composed of 5 questions about mental health to which the user has to answer about the frequency of the experience of some feelings in the previous four weeks. The range of punctuation is 1–6, 6 being the maximum emotional health quality index (Alonso et al., 1995). Short Form-36 Health Survey has shown high internal consistency [20].

Data collection and management: the information collected through the web link was deposited in an excel document from which the data could be exported to transform them into numerical data that allowed the calculation for statistical analysis.

Statistical analysis: The changes between the pre- and post-intervention measurements were studied. The non-parametric test for paired data, Wilcoxon’s test, was used. Statistical significance was established at a value of *p* < 0.05. To this end, the StataIC software program (version 16.1.824, College Station, TX, USA) was used.

## 3. Results

The description of the sample is displayed in Table 1. The median age of the ten caregivers was 48 years. Overall, 80% of women had a median care time of 6 years, 50% of them were spouses of the patients, and 60% of the patients were affected by stroke (hemorrhagic or ischemic cause). The main cognitive impairment of the patients reported by their relatives was memory deficit.

Table 2 shows a description of the basal emotional status of the caregivers. Overall, 50% of them presented an adequate level of emotional attention and emotional clarity, and 70% had an adequate (60%) or excellent (10%) emotional repair (TMMS-24). Moreover, 70% did not report overload (Zarit’s questionnaire), 40% showed an adequate (20%) or excellent (20%) resilience level, and the positive affect was higher than the negative affect in the previous month (Mdn = 32 vs. 20; PANAS). Finally, the median emotional health quality was 4.1 (1–6 range; MH5).

**Figure 3:**
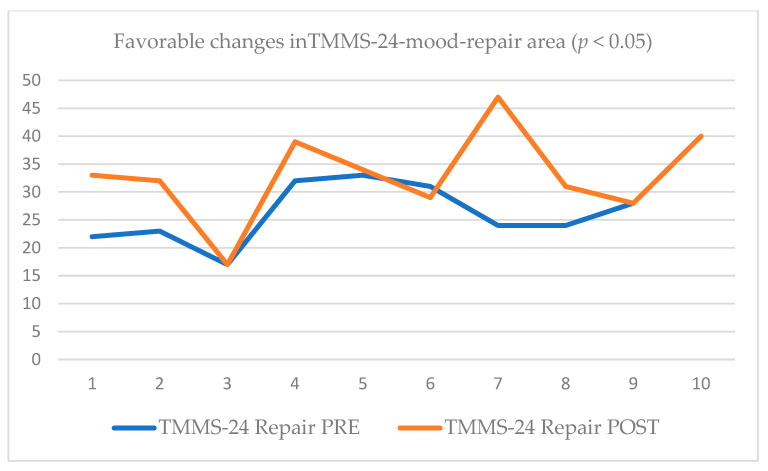
Pre-post scores of TMMS-24-mood-repair area.

After the training course, favorable changes related to emotional affect measured with the PANAS questionnaire were found; both positive (increase; Mdn = 39.5; effect size −12.79; adjusted variance 95.75) and negative (decrease; Mdn = 14.5; effect size 0.73; adjusted variance 95.50) presented a statistical significance of *p* < 0.05. The TMMS-24 post-test showed that 90% of the caregivers reported an adequate or excellent emotional repair (*p* < 0.05; effect size −0.68; adjusted variance 94.75; see Table 2 and Figure 3).

Nonsignificant differences after the program were found in the analysis of the rest of the variables: Caregiver Burden Interview (Zarit’s questionnaire), the 10-item Connor-Davidson Resilience Scale (10-item CD-RISC), and the Emotional Health Survey (MH5).

## 4. Discussion

The objectives of this study aimed to develop and evaluate a synchronous online training program on EI for the caregivers of adult patients with cognitive-behavioral impairment due to ABI. González-Fraile (2021) [21], in a Cochrane systematic review, remotely explored delivered interventions directed at the caregivers of dementia patients. They showed optimistic results, as there was a slight reduction in caregiver burden and improved depressive symptoms of the caregivers when compared with the provision of information alone, but not when compared with usual treatment, waiting list, or attention control. However, the cognitive impairment of a patient with dementia differs from that of a patient with ABI, not only in the degree and singularity of the affectation but also in the possibilities of improvement and prognosis. To our knowledge, there are no published results about the effectiveness of these types of interventions in caregivers of ABI patients in the scientific literature.

An ABI is a complex injury often followed by a broad range of physical, cognitive, emotional, and behavioral disabilities. There exists a bidirectional relationship between the well-being of the family and the patient [22]. It is essential to highlight that the physical and psychological health of the caregiver is often altered by the caregiving experience [23]. The situation is aggravated in the case of caregivers who are caring for someone with violent and/or disruptive behaviors. Specialized counseling programs could increase caregivers’ quality of life, which will ultimately affect the rehabilitation outcomes of persons with ABI [24]. A coherent understanding of the mechanisms underlying coping, adjustment, and resilience is needed to develop appropriate interventions for the caregiver population [25].

A meta-analysis of 20 studies found that when individuals have a greater ability to recognize their emotions and can perceive those of others, they have a better conflict management capacity [26]. Families have adaptive capacities that help them to establish effective psychosocial coping while living with patients with ABI [27]. A training program directed to reinforce these competencies can be seen as a helpful tool to improve their quality of life.

The situation related to the COVID-19 pandemic worsens caregivers’ situations. COVID-19 has required many countries across the globe to implement early quarantine measures as the fundamental disease control tool [28]. Isolation and the lack of normal routines have turned the care of a patient with cognitive impairment due to ABI into a more challenging task. Caregivers may feel fear of causing themselves and their relatives to be infected. This fear becomes acute when patients have risk factors for potential severe infections and, in addition, they are not completely aware of the safety measures. Previous outbreaks have reported that the psychological impact of quarantine can vary from fear, irritability, anger, frustration, loneliness, confusion, denial, anxiety, insomnia, despair, and depression [28]. A family without previous balanced emotional health could struggle in this challenging situation. Precisely, quarantine measures hinder face-to-face psychological training or group therapy to minimize the emotional impact. For this reason, the possibility of offering virtual options for counseling has to be considered an essential need.

Before our synchronous online training program on EI for the caregivers of adult patients with cognitive-behavioral impairment due to ABI, half of them showed low attention to their own emotions and low clarity or understanding of them, 30% of them showed low ability in emotional repair (measured with the TMMS-24). After the EI training course, 80% reported adequate clarity in emotional understanding, and 90% either adequate or excellent levels of emotional repair. The predominance of positive affect (pleasant emotions) versus negative was increased (32 positive and 20 negative in the pre-test vs. 39.9 positive and 14.5 negative in the post-test).

Our work is in line with Lehan (2016) [12], who highlighted shifting perspective from negative to positive aspects of the situation and learning to manage emotions as desirable objectives of counseling programs for caregivers of patients with TBI.

The improvement in emotional regulation found in our study could have great implications in light of the results of De-Torres (2022) [29]. They demonstrated that perceived EI strongly correlates to the mental health and resilience of caregivers. Additionally, they showed a relationship between the three concepts (EI, mental health, resilience) and an increase in the positivity of the emotions they experienced on a daily basis. They found that resilience, mental health, and emotional repair reduce overload and unpleasant emotions. Unpleasant emotions are associated with lower emotional repair, resilience, and mental health. In addition, overload increases daily unpleasant emotions.

Similar aspects are described in a study about primary caregivers of relatives with dementia (taking into account the clinical differences between dementia and ABI but assessing the similarities of the caregivers’ situation): it was demonstrated that those who have higher levels of resilience and EI spend more time on self-care and leisure activities [30]. There is also a recommendation for family support groups to assist the caregivers of mentally ill patients because it was found that there was a significant inverse relationship between the burden of care and psychological health [31]. Furthermore, it is a need recognized for professional caregivers [32], a population that has been found to experience burnout, which acts as a stressor and reduces the quality of care provided.

### Limitations

Our study is underpowered, with *n* = 10, which is a significant weakness. We must highlight the lack of a control group and the heterogeneity among participants related to gender, cause of ABI, age, and relationship to the patient. Further studies with a larger and more homogeneous sample and a control group should be performed to confirm our results. However, this study can be considered a starting point for future research.

## 5. Conclusions

After our training in EI, the caregivers of patients with cognitive impairment showed improvement in mood and management of unpleasant emotions. According to our data, the caregivers improved aspects of their EI, such as mood repair or emotional regulation, which are key abilities in people who not only have to practice self-regulation but are also in charge of the co-regulation of their patients. These results have to be considered with caution because of the quasi-experimental methodology, the sample size, and due to the lack of control over potential (external) intervening effects.

## Figures and Tables

**Table 1:** Sample characteristics.

Age of the Caregivers	Median 48 [21,62]
Gender of the caregivers	Two men (0.20)
	Eight women (0.80)
Number of years being a caregiver	Median, 6 [2,12]
Relationship with the patient	Two mothers of patient (0.20)
	Five spouses of patient (0.50)
	One sibling of patient (0.10)
	One daughter of patient (0.10)
	One niece of patient (0.10)
More dependent people on their charge	Two yes (0.20)
	Eight no (0.80)
Cause of brain injury of the patient	Three ischemic stroke (0.30)
	Three brain hemorrhage (0.30)
	Two traumatic brain injury (0.20)
	Two other causes (0.20)
Main cognitive deficit of the patient (observed by the caregiver)	Nine memory (0.90)
	One planification (0.10)

**Table 2:** Basal emotional status of caregivers and changes after the IE treatment.

	Pre-IE Training	Post-IE Training
TMMS-24 Attention	Low, 5 (0.50)	Low, 4 (0.40)
	Adequate, 5 (0.50)	Adequate, 5 (0.50)
	Excessive, 0 (0.0)	Excessive, 1 (0.10)
TMMS-24 Clarity	Low, 5 (0.50)	Low, 2 (0.20)
	Adequate, 5 (0.50)	Adequate, 8 (0.80)
	Excellent, 0 (0.0)	Excellent, 0 (0.0)
TMMS-24 Repair	Low, 3 (0.30)	Low, 1 (0.10) *
	Adequate, 6 (0.60)	Adequate, 6 (0.60) *
	Excellent 1 (0.10)	Excellent, 3 (0.30) *
PANAS-Positive affect	Median, 32	Median, 39.5 *
PANAS-Negative affect	Median, 20	Median, 14.5 *
Caregiver overload (Zarit’s questionnaire)	No overload, 7 (0.70)	No overload, 10 (1.0)
	Overload, 3 (0.30)	Overload, 0 (0.0)
	Intense overload, 0 (0.0)	Intense overload, 0 (0.0)
Resilience (CD-RISC-10)	Low, 6 (0.60)	Low, 3 (0.30)
	Adequate, 2 (0.20)	Adequate, 4 (0.40)
	High, 2 (0.20)	High, 3 (0.30)
Emotional health (MH5)	Median, 4.1 [1,6]	Median, 3.9 [1,6]

* Statistical significance (*p* < 0.05).

## Data Availability

Not applicable.
